# Genome Sequence of a Ranavirus Isolated from Pike-Perch *Sander lucioperca*

**DOI:** 10.1128/genomeA.01295-16

**Published:** 2016-11-17

**Authors:** Riikka Holopainen, Kuttichantran Subramaniam, Natalie K. Steckler, Sieara C. Claytor, Ellen Ariel, Thomas B. Waltzek

**Affiliations:** a Finnish Food Safety Authority Evira, Veterinary Virology Research Unit, Helsinki, Finland; b Department of Infectious Diseases and Pathology, College of Veterinary Medicine, University of Florida, Gainesville, Florida, USA; c Department of Wildlife Ecology and Conservation, University of Florida, Gainesville, Florida, USA; d College of Public Health, Medical and Veterinary Sciences, James Cook University, Townsville, Queensland, Australia

## Abstract

The pike-perch iridovirus (PPIV) was isolated in Finland from apparently healthy pike-perch fingerlings during routine disease surveillance. Our phylogenomic analysis revealed that PPIV is the first fish member of a clade of ranaviruses previously described from European and Chinese amphibians.

## GENOME ANNOUNCEMENT

Pike-perch iridovirus (PPIV) was isolated in Finland in 1995 from apparently healthy pike-perch fingerlings during routine disease surveillance (1). Internal organ homogenates of fish collected just before restocking resulted in cytopathic effects (CPE) on several fish cell lines, including bluegill fry (BF-2) and epithelioma papulosum cyprini (EPC) cells. Electron microscopy performed on infected BF-2 cell cultures revealed iridovirus-like particles. Infected cell cultures produced positive results for a direct immunofluorescent assay using rabbit polyclonal serum against several fish ranaviruses. Sequence analysis of the partial PPIV genome further supported its membership within the genus *Ranavirus* (2).

The PPIV isolate was amplified in EPC cells maintained in minimal essential medium (MEM) with 10% fetal bovine serum (FBS) at 23°C. Inoculation of EPC cells at a high multiplicity of infection (MOI) provided sixth-passage material harvested after 48 h when CPE was extensive. Cell culture supernatant was clarified at 3,000 × *g* for 20 min, and total nucleic acids were purified using a DNeasy blood and tissue kit (Qiagen). A DNA library was prepared using the Nextera XT DNA kit (Illumina), and sequencing was performed using a version 3 chemistry 600-cycle kit on a MiSeq platform (Illumina). *De novo* assembly of 4,981,658 paired-end reads in SPAdes (3) produced a contiguous consensus sequence of 108,041 bp, with a G+C content of 56.67%. The quality of the genome assembly was assessed by mapping the reads back to the consensus sequence in Bowtie 2 (4) and visually inspecting the alignment in Tablet (5). A total of 4,936,887 reads (99.1%) aligned at an average coverage of 12,082 reads/nucleotide.

A total of 109 putative open reading frames (ORFs) were predicted using GeneMarkS (6) and GATU (7) with common midwife toad virus (CMTV) isolates from the Netherlands (CMTV-NL; GenBank accession no. KP056312) and Spain (CMTV-E; GenBank accession no. JQ231222) used as reference genomes. Gene function was predicted based on BLASTP searches against the NCBI GenBank nonredundant protein sequence database. Comparative genomic analyses revealed that the aforementioned CMTVs and PPIV are very similar, except PPIV ORFs 7, 40, 50, 76, 81, 82, and 104 (hypothetical genes) were not included in one or both of the original CMTV genome annotations (8, 9). A frameshift mutation leading to an early stop codon was predicted in the CMTV-NL genome as compared to the complete ORF 76 (hypothetical protein) in PPIV and CMTV-E. Additional frameshift mutations leading to early stop codons were predicted in PPIV corresponding to ORFs 11 and 50 (hypothetical proteins) in CMTV-NL and CMTV-E.

Maximum likelihood phylogenetic analysis based on concatenated amino acid sequences of the 26 *Iridoviridae* core genes (10) revealed that PPIV belongs to a clade of ranaviruses isolated from European (CMTV) and Chinese *Andrias davidianus* ranavirus (ADRV; GenBank accession numbers KC865735 and KF033124) amphibians. An analysis of locally collinear blocks in Mauve (11) revealed that the PPIV, CMTV, and ADRV genomes are collinear.

Wild amphibians were observed in the same ponds that yielded PPIV from asymptomatic pike-perch, leaving open the possibility of interclass transmission (1, 12). Experimental PPIV infection trials did not generate disease in either pike-perch or rainbow trout; however, the virus was recovered from survivors in both studies, suggesting these hosts might serve as carriers (13). Other studies have demonstrated that PPIV can cause lethal infections in northern pike fry (14) and European common frog tadpoles (15), suggesting that the virus is capable of crossing host species barriers.

### Accession number(s).

The complete genome sequence of PPIV has been deposited in GenBank under the accession no. KX574341.
